# The Anorectic Actions of the TGFβ Cytokine MIC-1/GDF15 Require an Intact Brainstem Area Postrema and Nucleus of the Solitary Tract

**DOI:** 10.1371/journal.pone.0100370

**Published:** 2014-06-27

**Authors:** Vicky Wang-Wei Tsai, Rakesh Manandhar, Sebastian Beck Jørgensen, Ka Ki Michelle Lee-Ng, Hong Ping Zhang, Christopher Peter Marquis, Lele Jiang, Yasmin Husaini, Shu Lin, Amanda Sainsbury, Paul E. Sawchenko, David A. Brown, Samuel N. Breit

**Affiliations:** 1 St Vincent's Centre for Applied Medical Research, St Vincent's Hospital and University of New South Wales, Sydney, New South Wales, Australia; 2 Diabetes Research Unit, Novo Nordisk A/S, Maaloev, Denmark; 3 School of Biotechnology and Biomolecular Sciences, University of New South Wales, Sydney, New South Wales, Australia; 4 Neuroscience Program, Garvan Institute of Medical Research, Sydney, New South Wales, Australia; 5 The Boden Institute of Obesity, Nutrition, Exercise & Eating Disorders, Sydney Medical School, The University of Sydney, New South Wales, Australia; 6 Laboratory of Neuronal Structure and Function, The Salk Institute for Biological Studies, La Jolla, California, United States of America; INRA, France

## Abstract

Macrophage inhibitory cytokine-1 (MIC-1/GDF15) modulates food intake and body weight under physiological and pathological conditions by acting on the hypothalamus and brainstem. When overexpressed in disease, such as in advanced cancer, elevated serum MIC-1/GDF15 levels lead to an anorexia/cachexia syndrome. To gain a better understanding of its actions in the brainstem we studied MIC-1/GDF15 induced neuronal activation identified by induction of Fos protein. Intraperitoneal injection of human MIC-1/GDF15 in mice activated brainstem neurons in the area postrema (AP) and the medial (m) portion of the nucleus of the solitary tract (NTS), which did not stain with tyrosine hydroxylase (TH). To determine the importance of these brainstem nuclei in the anorexigenic effect of MIC-1/GDF15, we ablated the AP alone or the AP and the NTS. The latter combined lesion completely reversed the anorexigenic effects of MIC-1/GDF15. Altogether, this study identified neurons in the AP and/or NTS, as being critical for the regulation of food intake and body weight by MIC-1/GDF15.

## Introduction

MIC-1/GDF15 is a divergent member of the transforming growth factor-beta (TGFβ) superfamily [1], also known by a number of other names including growth differentiation factor 15 (GDF15) and non-steroidal anti-inflammatory drugs activated gene 1 (NAG-1). It circulates in the blood of normal individuals with a concentration range of about 150 to 1150 pg per ml [2], [3], however these levels rise in disease states. MIC-1/GDF15 is a stress response cytokine whose expression is further induced by processes such as inflammation, injury and malignancy [4], which also increase its serum levels [5]–[7]. Consequently, its circulating concentration represents a potential tool for the diagnosis and management of diseases such as premalignant colonic polyps, cancers, and cardiovascular diseases [3], [8]–[11]. Additionally, serum levels of MIC-1/GDF15 independently predict all cause mortality and cognitive decline in aging populations [12], [13].

In some disease states, especially in late stage cancer, chronic renal failure and severe congestive heart failure, MIC-1/GDF15 serum levels frequently rise to 10,000 to 20,000 pg per ml and occasionally to much higher levels [5], [14], [15]. Even moderate elevation in MIC-1/GDF15 serum levels decreased food intake in experimental animals, and prolonged elevation leads to cachexia [16]. High MIC-1/GDF15 serum levels may cause cancer anorexia/cachexia syndrome [17], [18], a frequently observed complication of advanced cancer, which causes significant morbidity, predominantly by limiting therapy and accelerating demise.

In addition to inducing anorexia in disease, recent data suggested that MIC-1/GDF15 might also play a role in the physiological regulation of appetite, body weight and fat mass [19], [20]. MIC-1/GDF15 germline gene knockout mice (MIC-1^−/−^) weighed more than their syngeneic controls, with females having a greater increase in weight than males. MIC-1^−/−^ mice of both sexes had an increased white fat mass, dominantly localised to visceral abdominal compartments. Female MIC-1^−/−^ mice also ate more and had reduced basal energy expenditure compared to syngenic controls, while male MIC-1^−/−^ mice had a milder phenotype. However, when male MIC-1^−/−^ mice were infused with sufficient recombinant human MIC-1/GDF15 (hMIC-1/GDF15) to raise their serum levels to the middle of the normal human circulating range, their body weight and food intake returned to the wild type level [19]. Consequently, It appears likely that the anorexia/cachexia associated with marked elevation of serum levels of MIC-1/GDF15, are due to the subversion of a physiological pathway for appetite regulation.

MIC-1/GDF15 is thought to exert its anorexigenic effects via direct actions on the central nervous system. Systemic administration has been associated with activation of neurons in the arcuate nucleus of the hypothalamus (Arc), the paraventricular nucleus of the hypothalamus (PVN) and in the area postrema (AP) of the brainstem [16]. In the Arc, where we have explored its effects in more detail, systemically administered hMIC-1/GDF15 induced anorexigenic proopiomelanocortin (POMC), while down regulating orexigenic neuropeptide Y (NPY) expression [16]. Further, viral-mediated MIC-1/GDF15 overexpression in the mediobasal hypothalamic nucleus caused profound anorexia and weight loss [16].

Like neurons in the Arc, brainstem neurons, especially in the AP and nucleus of the solitary tract (NTS) also play a key role in regulation of energy homeostasis [21]. Neurons of the AP and NTS can be activated by direct vagal afferent inputs from the gastrointestinal and other thoracic and abdominal viscera. In addition to synaptic inputs, blood-borne factors including circulating satiety hormones such as amylin, peptide YY (PYY) and glucagon-like peptide-1 (GLP-1) can also selectively alter neural activity in the AP and NTS. Here, they can enter the brain through the fenestrated capillaries in the AP [22], [23] and portions of caudal medial NTS [24], [25]. Additionally some molecules, including leptin, can be actively transported across the blood brain barrier (BBB) [26], [27]. Lastly, tanycytes found in both AP and NTS can also transport substances between blood and CSF [25].

Irrespective of how they are activated, neurons in the AP and NTS can transmit signals widely. AP and NTS neurons are densely and reciprocally interconnected and independently both have extensive reciprocal neuronal connections with the hypothalamus. These interconnections play an important role in translating signals from or to hypothalamic regions [28]–[30]. There are two major neuronal subtypes in the NTS: catecholamine (norepinephrine/epinephrine) containing neurons, which are commonly identified by the presence of its key regulator tyrosine hydroxylase (TH) and non-catecholaminergic neurons. These are composed of multiple subsets, each being identified by different, sometimes overlapping, markers such as glucagon like peptide-1 (GLP-1), POMC, cocaine and amphetamine regulated transcript (CART) or neurotensin [31]–[33]. Although both groups have been implicated in mediating anorectic effects, the actions of the catecholaminergic neurons in the NTS have been best characterized. For example, both cholecystokinin-8 (CCK-8) and leptin are known to activate catecholamine neurons in the A2/C2 region of the caudal medial NTS that project to the PVN oxytocin neurons [34], [35]. While non-catecholaminergic neurons within the NTS also play important roles in energy homeostasis [21], their functions are less well defined.

Neurons in the brainstem and especially in the AP and NTS play an important role in regulating energy homeostasis and are activated by MIC-1/GDF15 [16]. In this publication we focus our studies on characterizing which of these AP/NTS regions and neuronal subtypes are essential for the anorexigenic actions of disease-associated concentrations of MIC-1/GDF15.

## Methods

### Animals

All experiments were conducted in accordance with relevant guidelines and regulations with the oversight and approval of the Garvan/St Vincent's Animal Ethics Committee. Male C57BL/6 mice aged of 10–12 weeks were used, except where otherwise stated. All mice were housed under controlled temperature (22°C) with a 12:12 hr light-dark cycle (lights on at 07:00 hr) and had *ad libitum* access to a standard chow and water.

### Reagent and materials

Recombinant human MIC-1/GDF15 was expressed in *Pichia pastoris* and purified in house, in a 3-step process as previously described [36] [37]. Purified protein batches were tested and found to be free of endotoxin. Rabbit-anti-Fos antibody was generated and validated as previously described [38] and sheep-anti-tyrosine hydroxylase (AB1542) were purchased from Millipore (Merck KGaA, Darmstadt, Germany). DyLight™488-donkey-anti-rabbit IgG (711-475-152), and cy3-donkey-anti-sheep-IgG (713-165-287) were all purchased from Jackson ImmunoResearch (PA, USA).

### ICV administration of hMIC-1/GDF15

Mice, 11–13 weeks of age, with average weights between 25–30 g were anesthetized by intraperitoneal (IP) injection of ketamine-xylazine mixture (80 mg/kg, 50 mg/kg, respectively). The head was fixed to a stereotaxic platform (Model 900, KOPF instruments, Tujunga, CA) and a guide cannula (C315GS-4, Plastic One Inc., Roanoke, VA) was inserted through a burr hole into the right lateral cerebral ventricle, using the stereotaxic coordinates: −0.34 mm posterior from bregma; 1.0 mm lateral from bregma and depth at 2.4 mm from the skull [39]. The cannula was fixed to the skull with stainless steel screws and surgical glue (Cerebond, Plastics One Inc., Roanoke, VA). The cannula was capped (C315IDS-2, Plastics One Inc., Roanoke, VA) to avoid contamination and the mice were left to recover for 7 days. On the day of experimentation, an internal cannula (C315IS-4, Plastics One Inc., Roanoke, VA) was inserted through the guide cannula and connected to a Hamilton syringe (7102KH-2ul, Hamilton Reno., NV) with cannula tubing (C232CT, PE50/Thin wall, Plastics One Inc., Roanoke, VA). A total volume of 1 ul containing 50 ng of hMIC-/GDF15 or vehicle was then injected ICV slowly over 5 minutes.

### Immunofluorescence

To characterise MIC-1/GDF15 induced Fos immunoreactivity, mice were injected IP with hMIC-1/GDF15 (0.08 mg/kg) and anaesthetized at 30, 60 or 120 min post injection, prior to perfusion via the ascending aorta with saline followed by ice-cold 4% paraformaldehyde (PFA). Brains were removed and postfixed in 4% PFA at 4°C for 4 hr followed by cryoprotection in 30% sucrose in 0.1 M phosphate buffer (PBS) at 4°C for overnight. Coronal sections (30 µm) were cut on a sliding microtome through the whole rostro-caudal span of the NTS from the point where the NTS buds off from the dorsomedial edge of the spinal trigeminal nucleus to spino-medullary junction at the level of the pyramidal decussation (−3.40 mm to −4.44 mm from interaural line) [39], and stored in cryoprotectant (30% ethylene glycol, 20% glycerol, 50% 0.2 M PBS) until further processing. Immunofluorescent staining was performed in all sections. After washing in 0.1 M PBS, sections were incubated in rabbit-anti-Fos antibody diluted at 1∶10,000 in incubation buffer (0.1 M PBS containing 0.1% triton-X100 and 2% normal donkey serum) at 4°C for 48 hr. Sections were then washed 3 times with PBS before incubation in DyLight™488-donkey-anti-rabbit IgG (1∶200) at room temperature (RT) for 2 hr. followed by 3 washes in PBS. The sections were then mounted on glass slides and cover-slipped in 50% glycerol/PBS.

For dual labeling immunofluorescence, sections were incubated for 48 hr at 4°C in incubation buffer containing rabbit-anti-Fos at 1∶10,000 and sheep-anti-tyrosine hydroxylase at 1∶2000. Immunoreactivity was detected by incubating in DyLight™ -anti-rabbit IgG and cy3-anti-sheep IgG (1∶200) for 2 h at RT followed by 3 washes with PBS.

### Ablation of the AP and NTS

Ablation of AP and NTS were undertaken by the aspiration method [40]–[42]. The major advantage of aspiration lesioning is that it is done under direct vision, which ensures accuracy. Alternative strategies require stereotactic approaches, which are more difficult and less reproducible. Briefly, mice were anesthetized with 50 mg/kg ketamine (Pfizer, New York, NY), 25 mg/kg xylazine (Vedco, St. Joseph, MO) and fixed on a stereotaxic instrument (David Kopf Instruments, CA) with nose clamp and incisor bar. The incisor bar was lowered 6.15 mm below the interaural line to optimize AP/NTS access. A 1.5 cm longitudinal incision was made and the dorsal neck muscles retracted to visualize the atlanto-occipital membrane. The cisterna magna was entered by dividing the dura. In sham-ablated mice, no further surgery was undertaken. For AP ablation, the AP was visualized under the dissecting microscope and lesioned by aspiration, using a blunt 25 G needle connected to a vacuum pump drawing 10–12 psi. For AP and NTS ablation, aspiration area was extended bilaterally from the AP. Neck muscles were then re-apposed and the skin closed using stainless steel clips. After surgery, mice were given gel food (DietGel Recovery, Clear H2O, ME Portland) and 400 µl of saline subcutaneously every 12 hours for 4 days, then daily until their weight returned to 70–80% of that measures preoperatively. A minimum of 2–3 weeks recovery was allowed before proceeding with experiments. Of total of 36 mice underwent surgery, 4 died during the recovery and 32 mice survived, and experiment was commenced when their body weight returned to more then 85% of their preoperative weight.

### Histological assessment of AP and NTS ablation and cannula placement

Histological quantification of NTS lesioning and cannula placement was performed in all mice at the completion of each experiment by Nissl staining [43]. Mice were perfused with 4% PFA and brains were fixed and cryoprotected in 20% sucrose for overnight at 4°C. Brainstems were sectioned into 30 µm serial coronal sections then mounted onto glass slides. After air-drying, sections were labeled for Nissl material and the extent of the lesion was assessed under the microscope (DMI5500, Leica) [44]. In total of 32 mice that underwent surgery, 14 mice had complete ablation of AP with little or no damage observed in the NTS, and 12 mice had ablation confined to AP and medial NTS adjacent to the AP. In most cases, the ablated regions were from where the NTS adjoins the 4th ventricle and extended caudally to the level of obex. Maps identify the ablated regions are shown in the results. Of the 32 lesioned mice used for these experiments, 6 were not included in the data analysis, as at necropsy, lesions were found to be either too small or not confined to the region described above.

### Subcutaneous osmotic pump implantation

Seven-day-osmotic minipumps (model 1007D, ALZET Osmotic pump, Cupertino, CA) were loaded with vehicle or recombinant hMIC-1/GDF15 in a concentration that delivered 1 mg/kg/day at delivery rate of 0.5 ul/hr [19]. hMIC-1/GDF15 or vehicle-loaded pumps were implanted subcutaneously in the interscapular region of the AP or AP plus NTS ablated mice. Briefly, animals were anesthetized by inhalation of isoflurane then shaved and disinfected over the implantation site. A small incision was made across the midline and slightly posterior to the scapula, then a hemostat was used for blunt dissection to create a subcutaneous space for the pump, which was inserted with the delivery portal oriented caudally. The wound was closed with two 9 mm wound clips.

### Quantification of serum hMIC-1/GDF15 levels in mice

To determine the peak serum concentrations of hMIC-1/GDF15, mouse blood samples were collected at 0, 15, 30, 45, 60, 90, 120 and 240 minutes after a single IP injection of 0.08 mg/kg of hMIC-1/GDF15. To measure the steady state serum concentrations of hMIC-1/GDF15, blood sample were collected from mice at day 6 of infusion of hMIC-1/GDF15 via osmotic minipump. In both cases, blood samples were collected by cardiac puncture and allowed to clot by standing for 2 hr at 4°C. After centrifugation, the collected sera were stored at −70°C for assay of hMIC-1/GDF15, using a previously described in house ELISA [2], [45].

### Analysis of body weight and food intake in mice

Mice were single housed and acclimated on paper-towel (Chux, Padstow NSW Australia) bedding for 3 days prior to pump implantation and commencement of food intake. After the pump was implanted, body weight, food spillage on the bedding and food in the food hopper were weighed every morning between 9:00 to 10:00 hr for 5 consecutive days. Food consumption was determined by subtracting food left in the hopper and the spillage collected from the bedding, from the weight of the food supplied [16].

Since AP ablation itself leads to an increase in basal food intake [46], weight gain and food intake for the individual ablation groups were corrected by normalizing hMIC-1/GDF15 induced weight and food intake change to the average weight and food intake change from their respective vehicle treated control group. This was done by defining the mean body weight change in the vehicle treated arm of each group as 100%, and then scaling the weight change in the hMIC-1/GDF15 group appropriately (5C and 5D).

### Data analysis of immunofluorescent labeling

Five animals from each treatment group were analysed for Fos immunofluorescent staining and 3 animals from each treatment were analysed for dual Fos and TH staining. Very few Fos immunoreactive neuronal bodies were found in the rostral level of NTS that extends rostrally from the junction between the AP and the 4th ventricle to the NTS adjacent to the dorsomedial edge of the spinal trigeminal nucleus. Similarly, only very few Fos immunoreactive neurons were found in the caudal level of NTS that extend caudally from obex to the spino-medullary junction at the level of the pyramidal decussation. Therefore the immunoreactive neurons in the AP and the adjacent medial NTS were enumerated. Images were obtained and archived using a DMI6000B imaging microscope (Leica, Wetzlar, Germany). Counts were corrected using the Abercrombie correction equation for estimating numerical neuronal densities *P = A[M/(M+L)]* (von Bohlen und Halbach and Unsicker 2003; von Bohlen und Halbach et al. 2004). In this equation *P* is the average number of nuclear points per section; *A* is the crude counted number of nuclei seen in the section; *M* is the mean thickness of the virtual section; *L* is the mean length of the nuclei. In a first step, *A* is the counted in the different brain nuclei. In a second step, *L* was estimated for each different brain nucleus by computer-controlled measurement of the height of the cells in the *z* axis using the DMI6000B microscope.

### Statistical analysis

Statistical analyses were performed using GraphPad Prism software. A two-tailed unpaired t-test was used to assess significance in all cases unless otherwise specified. The threshold for statistical significance was set at *p*<0.05. All data are expressed as mean ± s.e.m.

## Results

### Intracerebroventricular administered hMIC-1/GDF15 induces anorexia and weight loss

**Figure 1:**
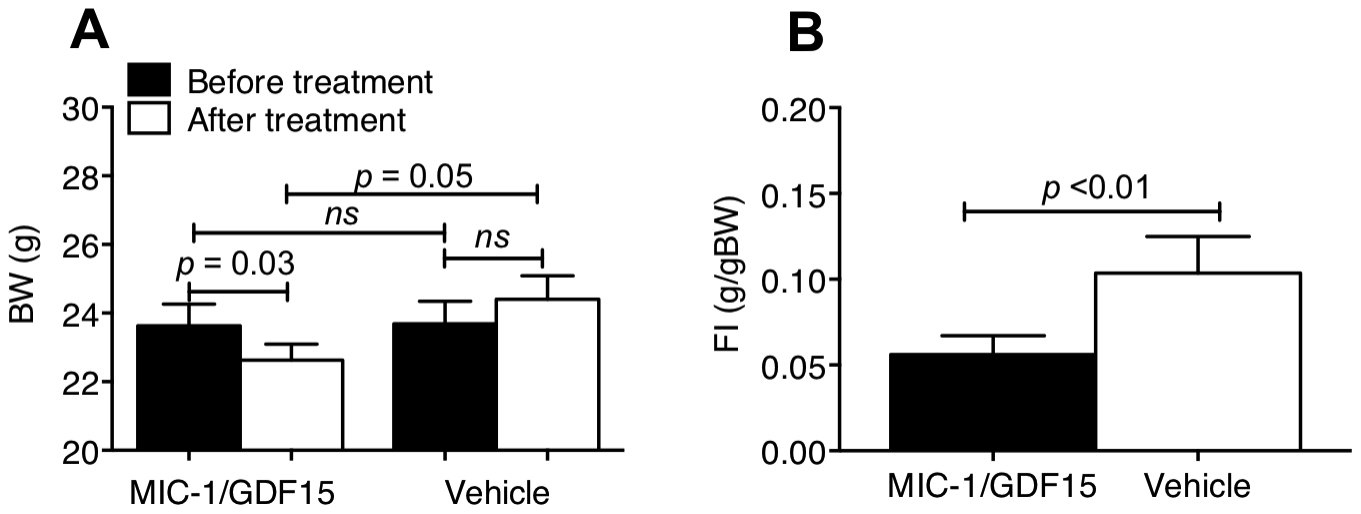
ICV administrated hMIC-1/GDF15 induces anorexia and weight loss. A single injection of 50-1/GDF15 or vehicle was delivered ICV. Changes in body weight (A) and changes in food intake (B) were compared 16 hr post injection. Data expressed as mean ± s.e.m. Abbreviations: BW, body weight; FI, food intake.

To provide further data supporting MIC-1/GDF15's direct central actions, and to complement previous studies in which we have induced local expression of MIC-1/GDF15 in the hypothalamus [16], we administered hMIC-1/GDF15 (50 ng) or vehicle directly to the CNS, via Intracerebroventricular (ICV) injection into the lateral ventricle, from where it flowed into the 3rd and 4th ventricles. hMIC-1/GDF15 treated mice lost an average of 1±0.2 g of body weight (*p* = 0.03, n = 6, paired t-test), while vehicle treated mice gained average of 0.5±0.2 g of body weight (*p* = 0.11, n = 6, paired t-test) (Fig. 1A). hMIC-1/GDF15 treatment effectively reduced the body weight in comparison to vehicle treated animal (hMIC/GDF15 vs vehicle, *p* = 0.05, n = 6). The weight loss in the hMIC-1/GDF15 treated group was due to reduced food intake as this group consumed significantly less food then the vehicle treated group (Fig. 1B) (*p* = 0.002, n = 6, unpaired t-test). To verify that the effects of ICV administered hMIC-1/GDF15 were not due to its access to the systemic circulation, a further two groups of 6 mice each were given either a single IP injection of vehicle or hMIC-1/GDF15 (100 ng) at double the dose given ICV. This systemic dose had no effect on body weight or food intake (data not shown). These data provide further strong support for the direct action of MIC-1/GDF15 on central appetite regulatory pathways.

### Systemic hMIC-1/GDF15 induces Fos immunoreactivity in AP, NTS, and caudal DMX

We have previously demonstrated a single IP injection of hMIC-1/GDF15 induces Fos immunoreactivity in mouse caudal brainstem neurons [16]. To determine the time of maximum Fos immunoreactivity induced by hMIC-1/GDF15, we gave groups of 5 mice a single IP injection of 0.08 mg/kg of hMIC-1/GDF15 or vehicle. Their brains were collected at 30, 60 or 120 minutes post injection, after which we mapped Fos immunoreactivity throughout the rostro-caudal span of the brainstem. This dose of hMIC-1/GDF15 induced peak hMIC-1/GDF15 serum levels to an average of 13.8±1.5 ng/ml (n = 8) at 30 minutes post injection. Prolonged steady state serum levels of this magnitude, are strongly associated with anorexia/cachexia in both mouse and man [16], [18].

**Figure 2:**
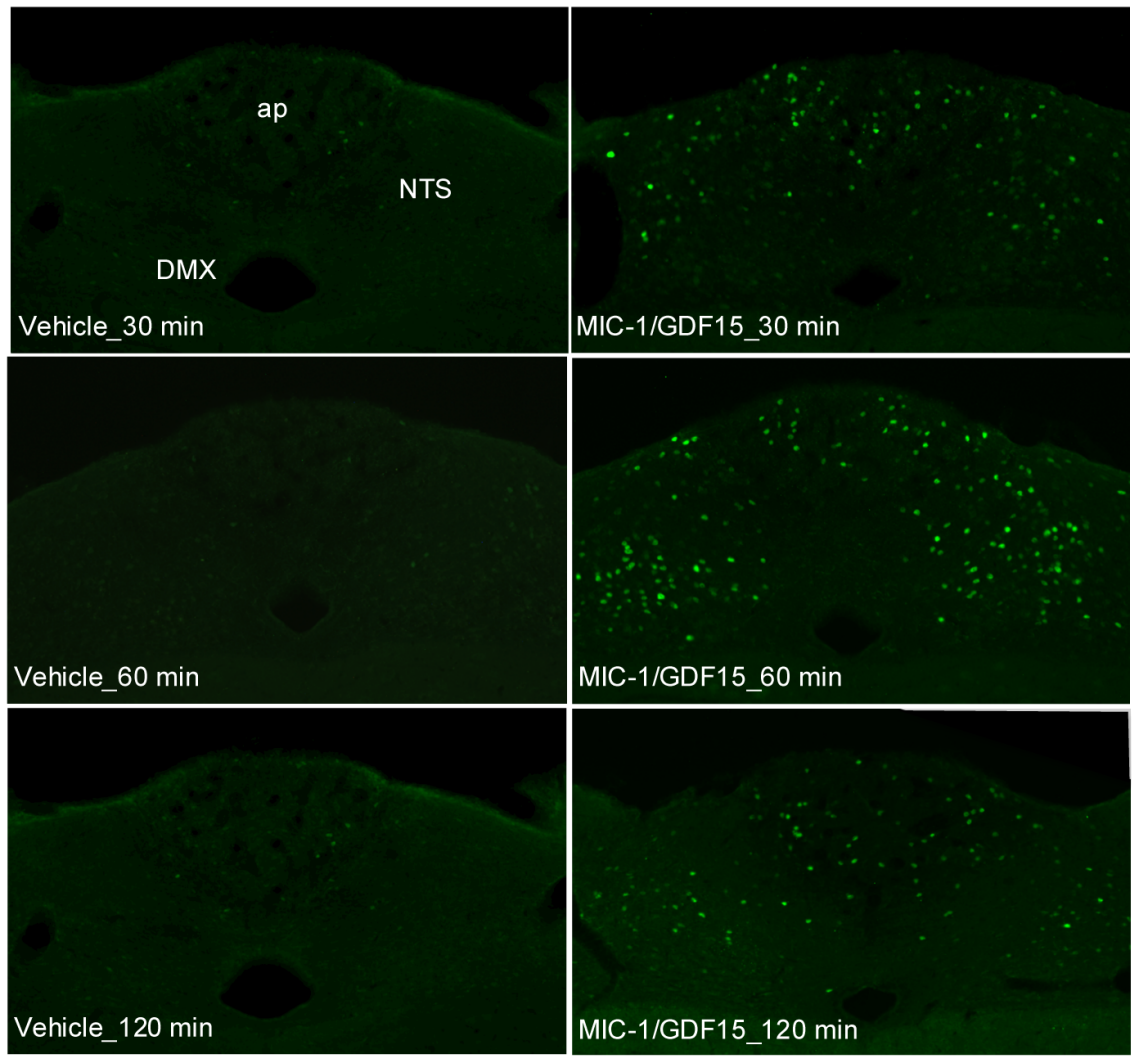
Systemic hMIC-1/GDF15 induces Fos immunoreactivity in brainstem nuclei. Photomicrographs represent coronal sections (−3.5 mm from interaural line) of Fos immunoreactivity at 30, 60 or 120 min after systemic IP administration of a single dose of hMIC-1/GDF15 or vehicle. Scale bar: 40 µm. Abbreviations: DMX, dorsal motor nucleus of vagus; ap, area postrema; cc, central canal; NTS, nucleus of the solitary tract.

For both AP and NTS, maximal Fos immunoreactivity was observed at 60 minutes after injection (Fig. 2). By contrast, injection of vehicle resulted in virtually no Fos immunoreactivity (Fig. 2). The distribution and density of activated neurons was consistent among individual hMIC-1/GDF15 injected animals (n = 5). The highest density of hMIC-1/GDF15 induced Fos immunoreactivity was observed in the AP and the medial division of NTS, especially in the intermediate, commissural subnuclei and subpostrema subnuclei of the medial NTS. Only few neurons with Fos immunoreactivity were observed at caudal level of NTS, which extends into the medulla to the junction with the spinal cord. Fos immunoreactivity was rarely found in the rostral NTS, from the level of the NTS at the junction between the AP and the 4th ventricle to the point where the NTS buds off from the dorsomedial edge of the spinal trigeminal nucleus. In the AP, the medial-caudal portion contained a higher density of Fos immunoreactivity. Additionally, hMIC-1/GDF15 induced Fos immunoreactive neurons were also observed in the caudal division of the dorsal motor nucleus of vagus (caudal DMX) extending from the level of obex to the spino-medullary junction at the level of the pyramidal decussation. No neuronal activation was seen in the rostral part of the DMX (i.e. caudal to the AP).

### MIC-1/GDF15 activates TH-positive and TH-negative brainstem neurons

**Figure 3:**
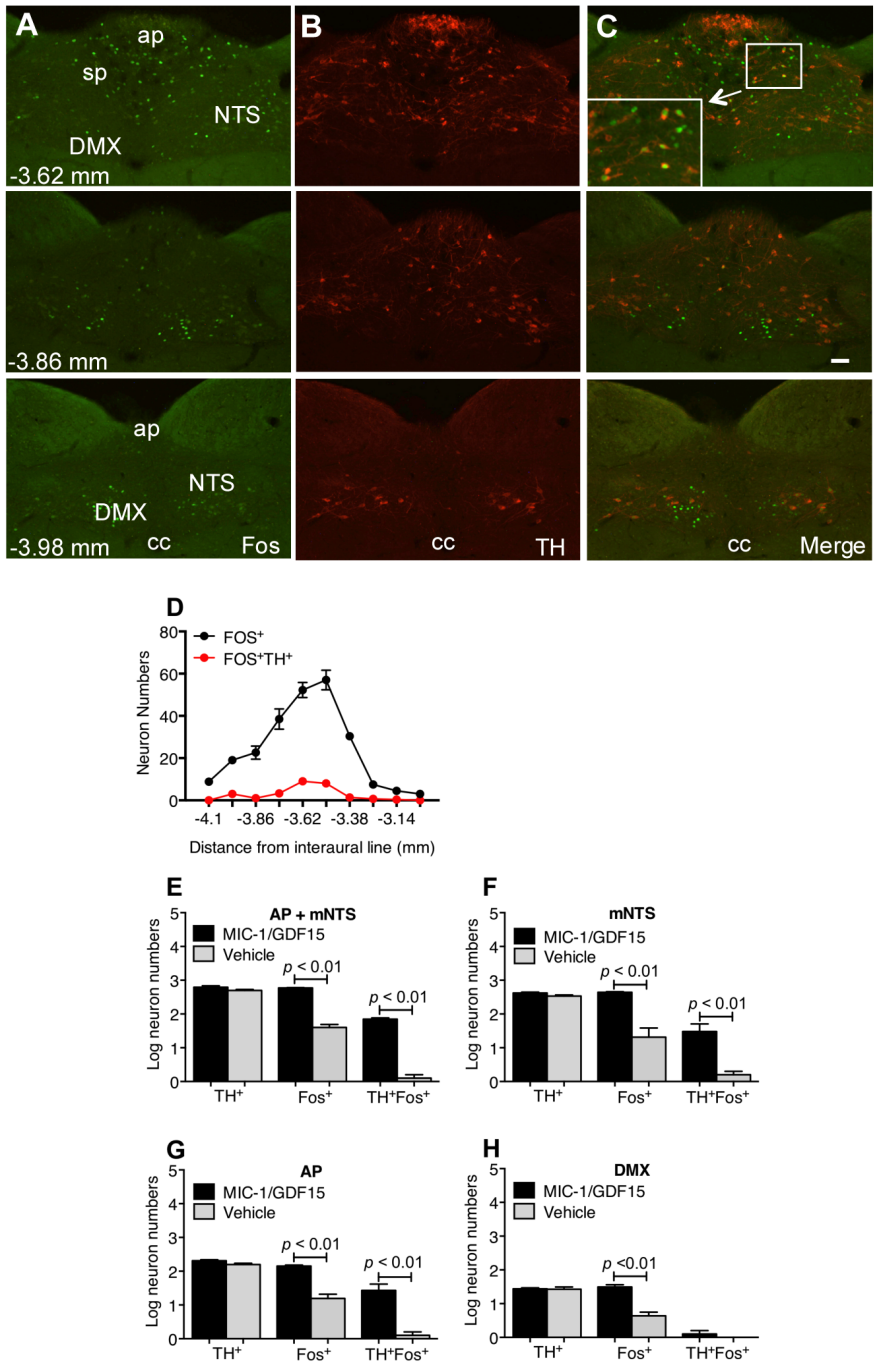
MIC-1/GDF15 activates both TH-positive and TH-negative neurons. Photomicrographs of coronal sections through the rostro-caudal span of NTS, demonstrate the distribution of dual Fos (Panel A, green), and TH immunoreactivity (Panel B, red). The merged images are in panel C, scale bar: 40 µm. (D) The Line graph shows the number of neurons that are Fos-positive or Fos and TH-positive in various AP/mNTS regions, identified as mm distance relative to the interaural line. (E-H) The bar graphs show the number of neurons that are immunoreactive to TH, or Fos, or both TH and Fos in mNTS, AP or DMX. Data are presented as mean ± sem. Abbreviations: DMX, dorsal motor nucleus of vagus; ap, area postrema; sp, subpostrema; cc, central canal; NTS, nucleus of the solitary tract; mNTS, medial region of nucleus of the solitary tract.

To characterize the pattern and phenotype of brainstem neurons activated by systemic administration of hMIC-1/GDF15, we carried out dual immunofluorescent staining for Fos and TH at 60 minutes after a single IP injection of (0.08 mg/kg) of hMIC-1/GDF15 (Fig. 3A, 3B, and 3C). Since hMIC-1/GDF15 predominantly activated neurons in the AP and the medial division of the NTS, quantitative analysis of staining was performed on these sites (Fig. 3D). Consistent with previously published reports [47], TH-positive neurons were seen within the AP, caudal DMX, and commissural subnucleus of medial NTS in a similar distribution to those activated by hMIC-1/GDF15 (Fig. 3B). An average of 629±39 TH-positive immunoreactive neurons and 590±11 Fos-positive neurons were identified in the AP and medial NTS of hMIC-1/GDF15 treated mice (Fig. 3E, n = 3), of which about 10% or 61±6 neurons showed colocalisation. In contrast, vehicle treated mice (n = 3) had an average of 48±2 Fos-positive neurons and 500±37 TH-positive neurons of which less then 0.1% or 1.3±0.3 neurons were colocalised (hMIC-1/GDF15 vs vehicle n = 3, *p*<0.001). As 10% of the hMIC-1/GDF15 induced Fos immunoreactive neurons were TH-positive and 90% were TH-negative neurons (Fig. 3D), it was clear that catecholaminergic neurons formed a minority of hMIC-1/GDF15 activated neurons in the AP and medial NTS, with the majority being non-catecholaminergic. In the DMX, hMIC-1/GDF15 treatment also resulted in Fos immunoreactivity in 32±4 (n = 3) neurons compared to 5±1 in vehicle treated animals (Fig. 3H, n = 3; *p*<0.01), none of which colocalised with TH-positive neurons.

### MIC-1/GDF15-mediated reduction in body weight and food intake is dependent on neurons in the AP and/or NTS

**Figure 4:**
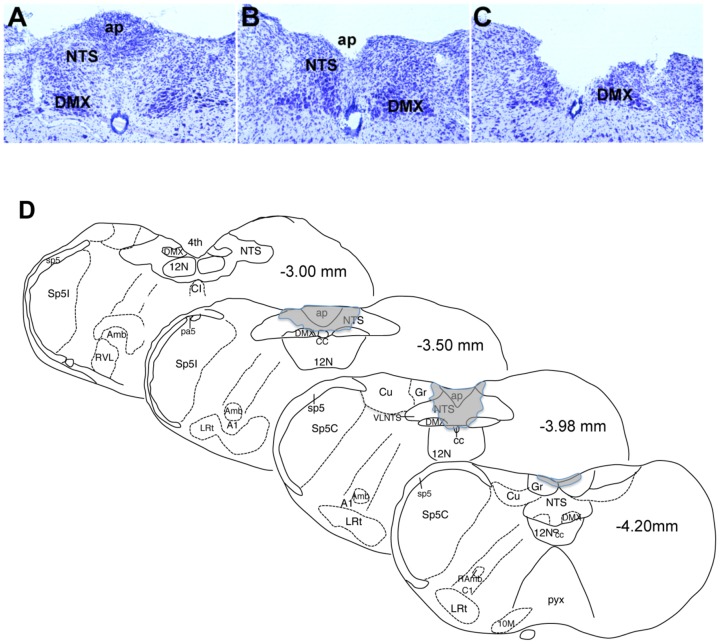
AP and NTS ablation. Representative Nissl stained sections of AP and mNTS from mice who were (A) Sham operated, (B) AP ablated or (C) AP/NTS ablated. (D) The extent of AP plus NTS lesioning is represented as shaded areas on drawings at four levels of the AP and NTS, which are localised by mm distances from the interaural line.

To determine the relative importance of the AP and NTS in the anorexigenic actions of disease-related concentrations of MIC-1/GDF15, we surgically ablated these regions in mice [42]. Aspiration lesioning of the AP alone removed all recognizable remnants of the AP, including the subpostrema in 14 mice, 6 mice of which were infused with hMIC-1/GDF15 and 8 with vehicle. Aspiration lesioning of the AP plus NTS was achieved in 12 mice, 7 of which were treated with hMIC-1/GDF15 and 5 with vehicle. The remaining NTS structures included the rostral NTS nuclei (extending rostrally from the level where NTS adjoins the 4th ventricle), caudal NTS (extending caudally from the obex), as well as some of the ventrolateral subnucleus of the medial NTS (Fig. 4D). Representative sections of sham operated, AP ablated and AP plus NTS ablated mice, labeled for Nissl material are shown in Fig. 4A–C respectively. The lesioned area overlapped very well with the region where hMIC-1/GDF15 induced Fos immunoreactivity was identified (Fig. 2 and Fig. 4). After recovery, mice were infused with vehicle or hMIC-1/GDF15 (1 mg/kg/day), which raised its steady state serum levels to an average of 0 ng/ml and 16.3±0.1 ng/ml (n = 8), respectively. The latter levels, if maintained, are strongly associated with anorexia/cachexia in mouse and man [16], [18].

**Figure 5:**
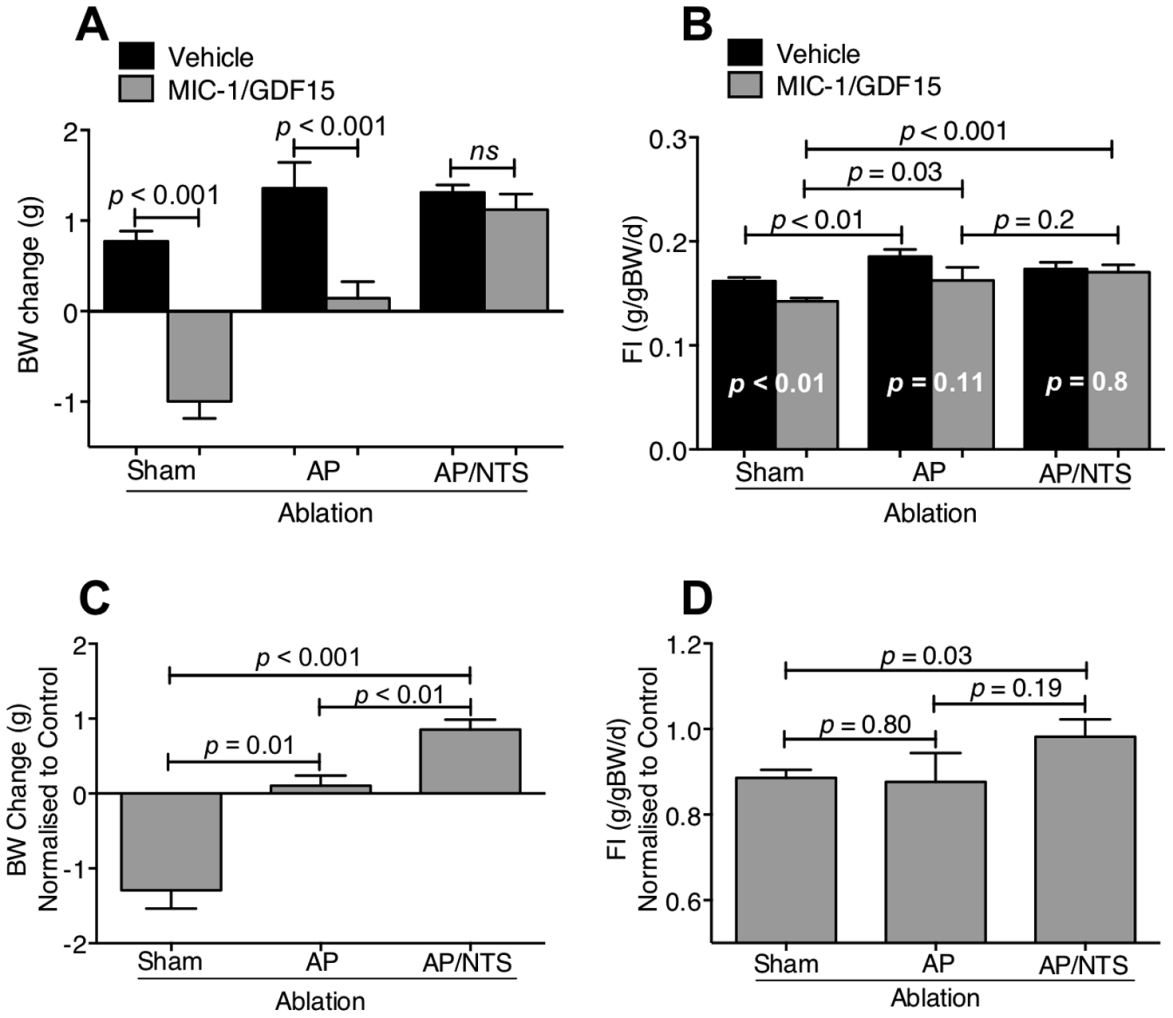
The effect of MIC-1/GDF15 on body weight and food intake reduction requires neurons in the AP and/or NTS. The effect of hMIC-1/GDF15 on mice (A) body weight change and (B) food intake change following ablation of the AP or AP plus NTS or sham surgery. The effect of MIC-1/GDF15 was normalised against the effect on vehicle treated mice for (C) changes in body weight and (D) changes in food intake. Data expressed as mean ± s.e.m. Abbreviations: BW, body weight; FI, food intake.

Five days of hMIC-1/GDF15 infusion induced significant weight loss in sham-operated mice, which lost an average of 1±0.2 g from their starting weight, while the vehicle treated animals gained an average of 0.8±0.1 g (Fig. 5A; hMIC-1/GDF15 vs vehicle; *p*<0.001, n = 5). Consistently, sham operated hMIC-1/GDF15 infused mice had a significant reduction in daily food intake compared to the vehicle treated mice (Fig. 5B; *p*<0.01, n = 5). This anorectic effect of MIC-1/GDF15 was completely abolished by AP/NTS ablation. Both vehicle treated mice and hMIC-1/GDF15 treated mice with AP/NTS ablation gained similar amounts of weight (respectively, an average of 1.3±0.1 g and 1.121±0.2 g; Fig. 5A; *p* = 0.6, n = 7). Food intake was also similar in these two groups (Fig. 5B; *p* = 0.8, n = 7). This indicated that an intact AP and NTS are required for MIC-1/GDF15 to exert its anorexigenic action.

By contrast, localized lesioning of the AP alone had only a small effect on the anorexigenic actions of MIC-1/GDF15. The AP ablated vehicle treated mice gained an average of 1.3±0.3 g from their starting body weight after five days of infusion. This was significantly different from AP ablated hMIC-1/GDF15 treated mice that had gained an average of only 0.14±0.02 g from their starting body weight (Fig. 5A; *p*<0.01, n = 6). AP ablated hMIC-1/GDF15 treated mice had significant weight gain compared to that of sham operated hMIC-1/GDF15 treated mice (Fig. 5A; *p*<0.01, n = 6), suggesting that AP ablation may have partially inhibited the anorectic effect of hMIC-1/GDF15. However AP ablated vehicle treated mice also ate more and gained significantly more weight compared to that gained by sham operated vehicle treated mice (Fig. 5A and 5B *p* = 0.02, n = 6; *p*<0.01, n = 6, respectively). Consistent with previous studies in rats, our vehicle treated AP ablated mice also displayed hyperphagia, suggesting a modification of control of food intake by the AP [46], which could potentially confound our comparisons between vehicle and hMIC-1/GDF15 treated AP/NTS ablated mice. Therefore, to account for this factor, weight gains for the individual groups were normalised as described in the methods. After this correction, a significant difference was found in the relative weight reduction in sham operated hMIC-1/GDF15 treated mice to that of AP ablated hMIC-1/GDF15 treated mice (Fig. 5C *p* = 0.01, n = 6). However, there was no significant change in normalised food intake between these two groups (Fig. 5D).

A similar normalization procedure was performed for the AP/NTS lesioned mice. hMIC-1/GDF15 infusion had no effect on either normalised weight change (Fig. 5C) or food intake (Fig. 5D) in this group, indicating that lesioning this area completely prevented the anorexigenic actions of hMIC-1/GDF15. This result differed significantly from the sham-operated control mice in which hMIC-1/GDF15 reduced food intake and body weight as expected (Fig. 5C–D. body weight *p*<0.001, n = 7; food intake *p*<0.03, n = 7).

## Discussion

This study reveals a key role for the brainstem in mediating the anorexigenic actions of MIC-1/GDF15. To explore its actions in the AP and NTS, we determined the distribution of neuronal Fos immunoreactivity in AP and NTS at 60 min after a single IP dose of hMIC-1/GDF15. This dose of hMIC-1/GDF15 activated mostly non-TH neurons in the AP and medial NTS. Ablating neurons in this region completely abolished hMIC-1/GDF15 induced effects on body weight and food intake, establishing neurons in the AP and/or NTS as essential for the anorexigenic actions of MIC-1/GDF15. However, exactly how MIC-1/GDF15 acts on these neurons is still unclear.

Neurons in the AP and/or NTS could directly respond to systemic MIC-1/GDF15 by binding to and activating the MIC-1/GDF15 receptor. While this receptor has not been identified, it is likely to belong to the highly conserved hetero-tetrameric chains of the TGF-b receptor (TBR) superfamily. Some data suggests that the TBRII, may form a component of the MIC-1/GDF15 receptor [16], [48], and this receptor is relatively highly expressed on AP neurons [49]. MIC-1/GDF15 is also known to activate the Arc and PVN, and this may occur indirectly via the activation of hindbrain neurons that project to these hypothalamic areas [50]–[52]. Alternatively, the AP/NTS might be activated via a multisynaptic route, initiated by the direct actions of MIC-1/GDF15 on the Arc. Finally, systemic MIC-1/GDF15 might act independently at both locations due to its direct actions via the fenestrated capillaries, which are a characteristic of these circumventricular organs.

In addition to the AP and NTS, systemic injection of hMIC-1/GDF15 also activated a small number of neurons in the caudal DMX. The major role of DMX neurons is to transmit nerve impulses through the vagus nerve down to the gastrointestinal tract. These caudal DMV neurons could be activated by systemic hMIC-1/GDF15-induced signals transmitted from the AP [24], [53]. Alternatively, they could be activated by synaptic input from NTS that carries signals originating in the vagus and other cranial nerves. Here MIC-1/GDF15, like CCK, might activate the vagus nerve to reduce gastrointestinal motility.

Only a small proportion of the hMIC-1/GDF15 activated neurons were TH-positive. This indicates that, unlike satiety factors such as CCK and oleoylethanolamide, which are released from the small intestine and activate NTS catecholaminergic neurons [54], [55], hMIC-1/GDF15 predominantly activated non-catecholaminergic neurons. However, this does not exclude a contribution of catecholaminergic neuronal activation to MIC-1/GDF15 effects. Further studies will be required to more precisely identify the major AP/NTS neuronal subsets, through which MIC-1/GDF15 acts.

Complete ablation of the AP/NTS complex completely inhibits the anorexigenic actions of MIC-1/GDF15 whilst ablation of the AP alone results in little inhibition. This suggests that the AP is likely to play a smaller part in MIC-1/GDF15 control of food intake. Further studies will require cell specific deletion of the MIC-1/GDF15 receptor but these will not be possible till it has been identified and characterized.

In sum, this study demonstrates that neurons of the NTS are critical for the anorectic actions of disease-associated concentrations of MIC-1/GDF15, an effect that may be dominantly mediated by activation of non-catecholaminergic neurons. It is yet to be determined if lower concentrations of MIC-1/GDF15, closer to its physiological levels, also act through the brainstem, or through other regions of the brain such as the Arc and/or PVN.
